# Silicon-Carbide (SiC) Nanocrystal Technology and Characterization and Its Applications in Memory Structures

**DOI:** 10.3390/nano10122387

**Published:** 2020-11-29

**Authors:** Andrzej Mazurak, Robert Mroczyński, David Beke, Adam Gali

**Affiliations:** 1 Institute of Microelectronics and Optoelectronics, Warsaw University of Technology, Koszykowa 75, 00-662 Warsaw, Poland; a.mazurak@imio.pw.edu.pl; 2 Wigner Research Centre for Physics, POB. 49, H-1525 Budapest, Hungary; beke.david@wigner.hu (D.B.); gali.adam@wigner.hu (A.G.); 3 Department of Atomic Physics, Budapest University of Technology and Economics, Budafoki út 8., H-1111 Budapest, Hungary

**Keywords:** silicon-carbide (SiC) nanocrystals, high-k dielectric, metal–insulator–semiconductor (MIS), metal–insulator–metal (MIM), electrical characterization

## Abstract

Colloidal cubic silicon-carbide nanocrystals have been fabricated, characterized, and introduced into metal–insulator–semiconductor and metal–insulator–metal structures based on hafnium oxide layers. The fabricated structures were characterized through the stress-and-sense measurements in terms of device capacitance, flat-band voltage shift, switching characteristics, and retention time. The examined electrical performance of the sample structures has demonstrated the feasibility of the application of both types of structures based on SiC nanoparticles in memory devices.

## 1. Introduction

Due to the potential applications in the field of modern electronic and photonic devices, the investigations and feasibility studies of the structures with nanocrystals (NCs) embedded in dielectric ensembles are commonly noticeable [1]. The literature mostly reports the application of silicon (Si) nanocrystals in various semiconductor devices and structures, e.g., field-effect light-emitting devices (FELEDs) [2], photovoltaic cells [3], memory structures based on photoluminescence (PL) [4], or non-volatile semiconductor memory (NVSM) devices [5,6]. Recently, the other types of semiconductor NCs or all-inorganic perovskite NCs and nanoparticles (NPs) have significantly attracted scientific interest [7].

This study presents the technology and characterization of memory structures that are based on cubic silicon-carbide (SiC) NCs. The fabrication of SiC-NCs was based on the reactive bonding method followed by electroless wet chemical etching [8]. In the next step, SiC nanoparticles were successfully incorporated into the technology of the metal–insulator–semiconductor (MIS) and metal–insulator–metal (MIM) structures. The benefit of the proposed technology of MIS and MIM devices with SiC-NCs, as compared to the typical formation of NC-based devices, is much easier embedding of NCs into the gate-stack; most importantly, the technology can be characterized as extremely low temperature, which follows the lowering of the thermal budget of modern semiconductor device technology [9]. Moreover, the transfer of SiC-NCs from the liquid solution to the semiconductor substrate is compatible with complementary metal-oxide-semiconductor (CMOS) technology since it can be done similarly to the photoresist spin-coating procedure. Test structures with and without NCs embedded in the stack were fabricated. The electrical measurements were performed, and the results were used for device parameters extraction. A comparative discussion of the results is presented, which reveals the essential difference between the structures with and without NCs, due to the nanoparticle charging and discharging processes. Although the presented results are preliminary, relatively large charge retention was obtained. The discussion proves the feasibility of applying the investigated MIS and MIM structures in memory devices, particularly in NVSM devices or resistive random access memories (RRAMs).

## 2. Silicon-Carbide (SiC) Nanocrystal Technology and Characterization

A bottom-up technique was used to synthesize cubic SiC powder from its elements [8,10]. Si (99%, 325 mesh, Sigma-Aldrich, St. Louis, MO, USA) and C (Norit A supra) with 1:1 ratio and 3 wt% PTFE (polytetrafluoroethylene) (Sigma-Aldrich, St. Louis, MO, USA) were mixed in a ball mill, then placed into a graphite crucible. Samples were annealed to about 1250 °C in an argon atmosphere for 8 min using an induction furnace. To introduce stacking faults in the cubic SiC matrix, 5 wt% Al powder (95%, <5 μm, Sigma-Aldrich, St. Louis, MO, USA) was added to the mixture mentioned above. SiC NPs were then fabricated from the SiC powder by the no-photon exciton generation chemistry (NPEGEC) technique [10,11].

The bulk cubic SiC was briefly etched in a mixture of hydrofluoric acid and nitric acid solution in an autoclave at 150 °C. The thin porous layer, which is the result of the NPEGEC stain etching, was broken into SiC NPs by ultra-sonication. The size distribution of the SiC NPs was controlled through the stacking faults (SF) in the cubic SiC. Aluminum was used as an additive to the mixture of silicon and carbon during SiC powder synthesis to vary the stacking fault content. The SiC NPs prepared from the cubic SiC with low SF concentration were smaller than 4 nm. Increasing the SF concentration increased the number of SiC NPs between 4 and 6 nm. In the case of the studied samples, 5 m/m% Al was used to achieve ca. 15% SF concentration, resulting in particles in the range of no more than 4–6 nm.

**Figure 1:**
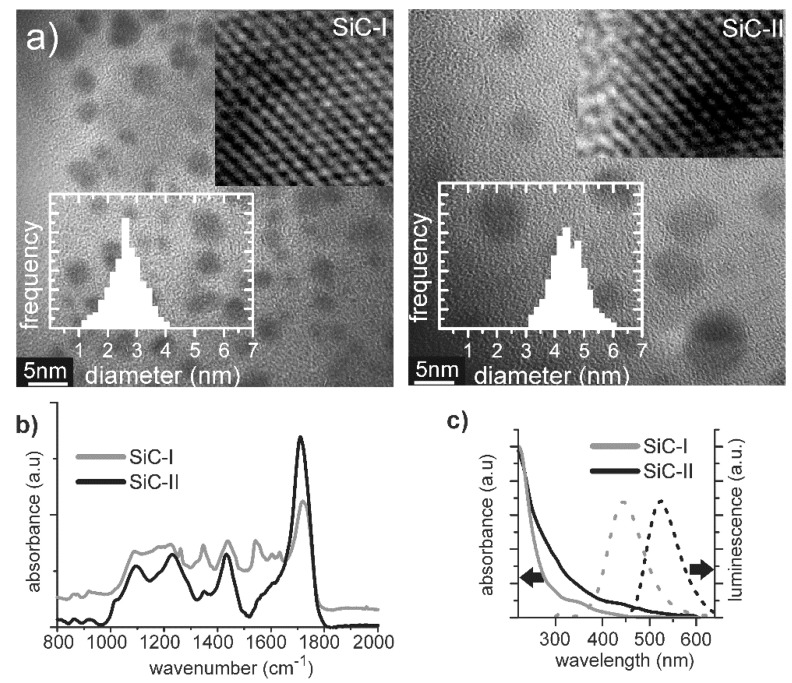
(**a**) High-resolution transmission electron microscopy (HR-TEM) images of as-prepared ultrasmall, 1–3 nm SiC (SiC-I), and larger, 4–6 nm (SiC-II) SiC, NPs with the corresponding size distribution; (**b**) FTIR spectra of SiC-I and SiC-II representing the surface termination of the particles; (**c**) UV-VIS absorption and photoluminescence spectra of SiC-I and SiC-II; from the UV-VIS and the photoluminescence excitation (PLE) spectra (not shown), one can calculate the optical bandgap of the NPs.

The size distribution and crystallinity level of the particles were studied by high-resolution transmission electron microscopy (HR-TEM, Figure 1a). The SiC NPs were deposited onto a TEM grid from an aqueous solution. According to the HR-TEM imagery, the SiC NPs were well crystalline without the detectable presence of an amorphous or oxide layer. Regardless of their sizes, the SiC-I and SiC-II NPs had an almost identical surface termination. Fourier transformed infrared spectra (FTIR, Figure 1b) of the samples were dominated by the C=O stretching vibration of the –COOH at around 1720 cm^−1^ and corresponding C–O stretching vibration at around 1200 cm^−1^. The spectra also showed a broad band centered at 1100 cm^−1^ that was assigned to C–O–C and Si–O–Si vibrations, as well as CH vibrations between 1400 and 1600 cm^−1^. The optical bandgap varies with size from 3.87 eV (1–3 nm ultrasmall NPs) to 2.85 eV (4–6 nm NPs) (Figure 1c) [11].

SiC-I and SiC-II nanoparticles shared the same surface termination. However, the difference in the bandgap originated from a different electron configuration that was influenced by the surface groups. In the case of 1–3 nm particles, the increase of the relative number of surface atoms contributing to the surface states created flat valance states or HOMO levels, and those were independent of the particle size. Such an effect was diminished for larger particles. As a result, SiC-I had size-independent, molecular-like properties, whereas the optical properties of SiC-II were strongly size-dependent [11]; the electron and hole transfer from SiC-II to a surrounding media was found to be significantly stronger [12] as well.

## 3. Sample Devices Fabrication and Measurement Protocol

**Figure 2:**
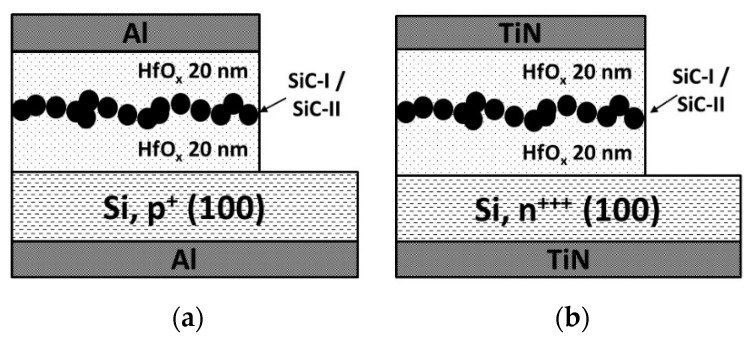
Sample devices fabricated for this study: (**a**) NC-MIS and (**b**) NC-MIM types.

**Figure 3:**
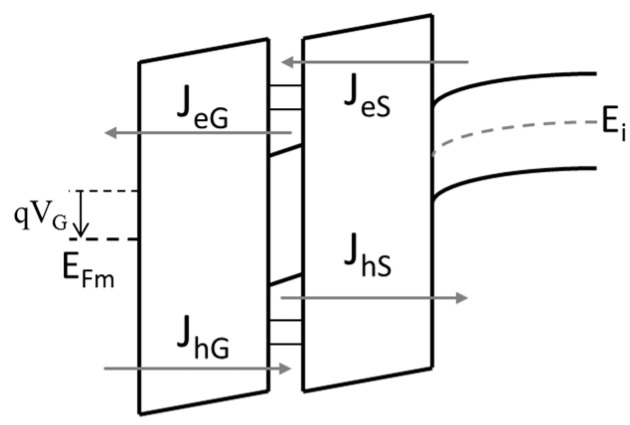
The schematic band diagram of the sample device.

The colloidal cubic silicon-carbide nanocrystals were introduced into metal–insulator–semiconductor (MIS) and metal–insulator–metal (MIM) structures based on hafnium oxide layers. Two types of sample devices were fabricated: NC-MIS (nanocrystal MIS) and NC-MIM (nanocrystal MIM), and they were accompanied by reference devices, i.e., without nanocrystals embedded in the insulator layer. The schematic cross-sections and the schematic band diagram of the sample devices are presented in Figure 2; Figure 3 respectively. The two types of investigated devices may, at first glance, seem to be very similar in Figure 2 and Figure 3; they differ essentially in terms of their operation principles. The sample NC-MIS devices were fabricated on silicon substrates with the resistivity of 1–10 Ωcm (boron-doped), whereas the NC-MIM devices used highly doped silicon substrates (the resistivity of 0.0010.002 Ωcm, antimuonium impurities). The information was stored in the NC-MIS structures in the form of an electric charge. The nanocrystals embedded in the gate-stack constituted a quantum well that stored the charge. The charge carriers (electrons and holes) were delivered or removed by the tunnel communication between the well and the gate electrode (*J_eG_*, *J_hG_*), or the well and the semiconductor substrate (*J_eS_*, *J_hS_*). In the case of the investigated NC-MIM structures, the highly doped substrate played the role of the bottom electrode. The stored information was represented by the resistance value extracted at the outer electrodes. The resistivity of the insulator layer that was encapsulated between the top and the bottom electrode could be modulated by creating and breaking conducting filaments. The nanocrystals embedded in the layer may have played the role of seeds for induced electrical current paths.

**Figure 4:**
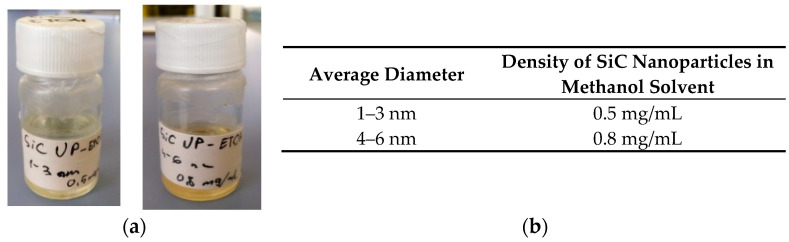
(**a**) Colloidal silicon-carbide nanocrystals (SiC-NCs) dispersed in methanol, and (**b**) specifics of the liquid solution.

The processing sequence of the fabricated structures was as follows: Si substrates were cleaned utilizing a modified RCA method (Piranha + SC1 + SC2 + HF dipping). In the first step, a 20 nm bottom hafnium oxide (HfO_x_) layer was deposited. In the next step, the SiC NPs spinning off on the top of the hafnia surface was performed, followed by 20 nm top HfO_x_ layer deposition. Two types of colloidal nanoparticles were used; those with the dimensions of 1–3 (SiC-I), and those measuring 4–6 nm (SiC-II) (Figure 4). After the formation of SiC-NCs embedded in dielectric layer ensembles, in the case of NC-MIS structures, the aluminum (Al) contact pads were formed through the standard UV (@365 nm) photolithography process and wet etching, whereas in the case of NC-MIM devices, titanium nitride (TiN) contacts were obtained using the “lift-off” procedure. The HfO_x_, Al, and TiN layers were deposited in the pulsed-DC reactive magnetron sputtering process. Post-metallization annealing (PMA) at 300 °C in a vacuum atmosphere was performed at the end of the processing sequence. The performed PMA process not only improved the contact properties of the processed sample structures, but also significantly enhanced the quality and electrical parameters of HfO_x_ films, as had been previously demonstrated [13,14].

**Figure 5:**
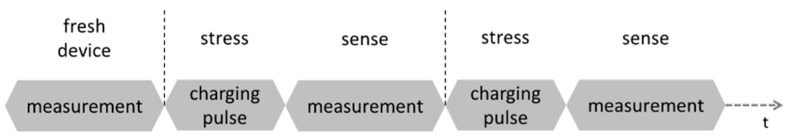
The measurement procedure which was implemented during the electrical characterization of investigated structures.

The electrical measurements always started with measurements performed for a fresh device. Then, subsequent electrical pulses (i.e., the electrical stress) were imposed, followed by electrical measurements (i.e., the sense component of the procedure), as presented in Figure 5. The charging pulses were performed with a voltage or a current source. The measured parameters were the current (static measurements) or the small-signal capacitance (admittance measurements) conducted in the voltage or time-domain, i.e., in the sweeping or sampling modes, respectively. For the program-erase voltage investigations, subsequent voltage pulses of the opposite polarity were used. The electrical measurements were conducted with the Keithley 4200 semiconductor characterization system and SUSS PM-8 probe station, as presented in [15]. The procedure of electrical parameters extraction used in this work was described in detail in [16]. The results presented in this study were obtained for NC-MIS and NC-MIM devices, with a top electrode area of 1.8 × 10^−4^ cm^2^.

## 4. Results and Discussion

**Figure 6:**
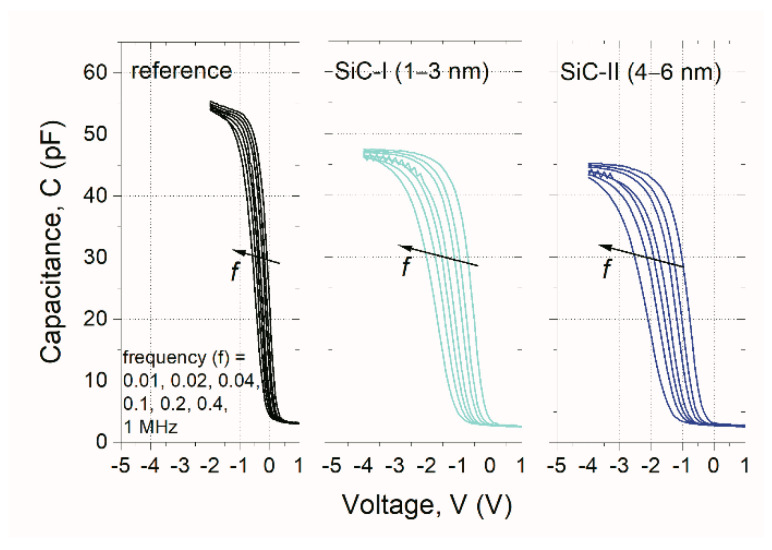
Obtained high-frequency families of C–V curves of examined in this work MIS structures; f = 0.01, 0.02, 0.04, 0.1, 0.2, 0.4, 1 MHz.

The results depicted in Figure 6 show the high-frequency C–V curves of NC-MIS structures with SiC nanocrystals as compared to the reference device. The noticeable different quantity of frequency dispersion within the curves suggests that the built-up charge in the examined structures with NCs can be observed. Such an effect can be noticed for both dimensions of SiC NPs, which is due to the introduction of NCs in the hafnia films and the formation of the quantum well that allowed for the charge carriers trapping. Another effect that can be also noticed is the lowering of the maximum capacitance value with the increase of NCs size in the accumulation regime due to nanocrystals embedded in HfO ensembles.

**Figure 7:**
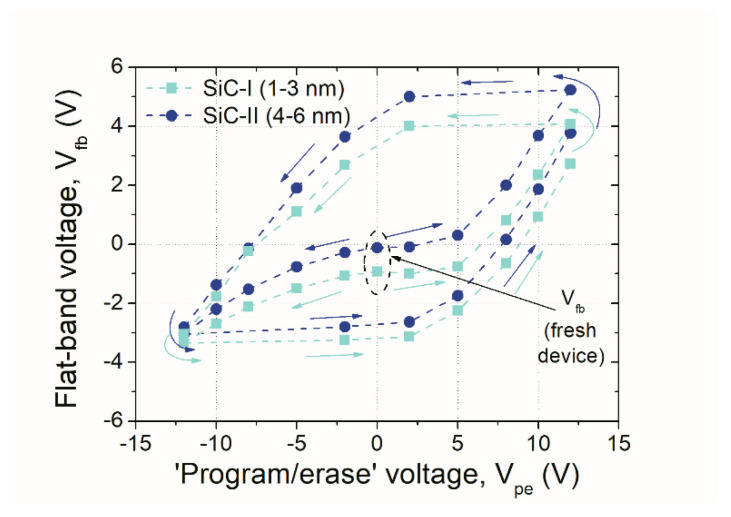
Memory window (expressed as V_fb_ change) of NC-MIS devices with both types of SiC-NCs examined in this study; V_p/e_ = 0–±12 V, and stress time = 1 s were used.

The memory effect in the examined NC-MIS devices was investigated by determining the flat-band voltage (V_fb_) shift revealed from the C–V characteristic after applying specific voltage stress. The V_fb_ value can be treated as the distinctive parameter of the MIS device, similar to the threshold voltage (V_th_) value of the MOSFET. Furthermore, the examinations of V_fb_ changes of the MIS device at specific stress values can be valuable for memory effects characterization [17]. Based on the V examination from the C–V curves after stressing (i.e., after excitation at specific voltage value), the hysteresis loop of the examined NC-MIS device V_fb_ = f(V_p/e_) was obtained; this is presented in Figure 7. Such a hysteresis can be considered as the memory window of the examined test device.

In the case of the NC-MIS structure with SiC-I NPs, a change of V of the order of 7.6 V was obtained, whereas in the case of devices with SiC-II NPs, the obtained memory window of the order of 8.6 V was observed. However, it is worth noting that in the case of the NC-MIS structure with SiC-I NPs, the symmetry of maximum and minimum values of V after the positive and negative V around the flat-band voltage of a “fresh” device, i.e., the MIS structure without any program or erase procedure is increased. Such a finding allows for a more convenient implementation of the examined structures in a specific memory application.

**Figure 8:**
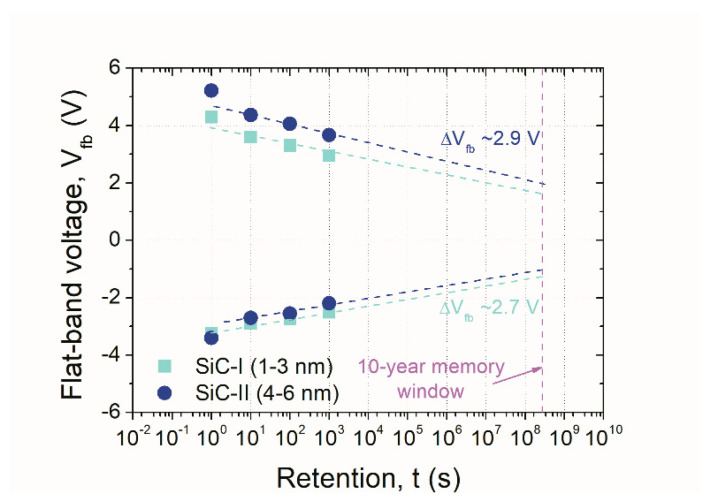
Memory window (expressed as V_fb_ change) of NC-MIS devices with both types of SiC-NCs; V_p/e_ = 0–±12 V, and stress time = 1s were used.

A further performed examination of the permanence of introduced charge into examined NC-MIS structures confirmed the large charge retention. The results of retention investigations are collected in Figure 8. The obtained retention of the V shift extrapolated to ten years exhibited similar persistence of charges for both types of the investigated sample devices (i.e., SiC-I and SiC-II NCs). In the case of NC-MIS structures with smaller NCs (i.e., 1–3 nm), the charge loss was of the order of ~64%, whereas in the case of larger NPs (i.e., 4–6 nm), the charge loss was increased to ~66%. Those values corresponded to the memory windows width of the order of 2.7 V and 2.9 V for NC-MIS structures with SiC-I and SiC-II nanocrystals (extrapolated to 10 years).

**Figure 9:**
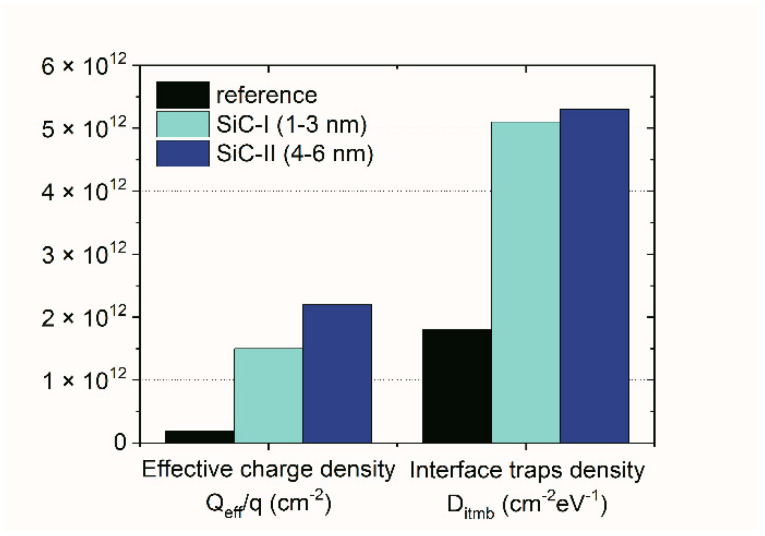
Extracted parameters showing the effective charge density (Q_eff_/q) and interface traps density in the middle of the silicon bandgap (D_itmb_) for NC-MIS structures and reference MIS devices investigated in this work.

The observed effect of the slightly wider memory window and higher retention loss for NC-MIS structures with a larger dimension of SiC-NCs compared to structures with SiC-I NPs was further investigated. The basic electrical parameters estimated from the C–V curves were calculated. Figure 9 depicts the effective charge density (Q_eff_/q) and the interface trap density in the middle of the silicon bandgap (D_itmb_) for the NC-MIS devices examined in this study. It has to be taken into account that the estimated electrical parameters represent the “effective” values for the whole gate-stack.

As shown, both Q_eff_/q and D_itmb_ values were higher for MIS structures with SiC-NCs than the reference device. Due to the presence of nanoparticles embedded in the HfO_x_ layer bulk, the results were also proved by the comparison depicted in Figure 6. It is also evident that the effective charge was about one third higher for the structure with SiC-II compared to NC-MIS devices with SiC-I, which resulted in a higher density of charge that may have been trapped in the gate-stack of the examined structure, explaining the higher memory window (i.e., 2.9 V) reported above. Simultaneously, the slightly higher D value that was obtained for the NC-MIS structure with SiC-II may have been the cause of higher leakage current (sequential tunneling process via the interface and border traps) and a higher probability of charge loss from the NCs film that was embedded in the HfO_x_ insulator layer. As the interface trap density directly affected the leakage current [18], it is a reason for a slightly higher retention loss that has been observed for NC-MIS devices with SiC-II NCs (compare the retention loss of 64% vs. 66% for NC-MIS SiC-I and NC-MIS SiC-II, respectively).

The literature demonstrates several examples of applications of structures with nanocrystalline islands in memory devices. These exemplary devices are mainly established on Si nanocrystals introduced in silicon dioxide (SiO_2_) films. The reported memory windows of these devices differ depending on such factors as the studied type of a gate structure, the conditions of electrical polarization, and stressing pulses. As an example, the devices were deposited using the low-pressure chemical vapor deposition (LPCVD) method; thermally recrystallized Si-NCs that were introduced in SiO_2_ can be characterized by the memory window manifested as the change of V _fb_ under V _p/e_ of the order of 1.6 V and 3.5 V, for a bipolar asymmetric and unipolar sequence of program and erase pulses, respectively [19]. In the case of Si clusters obtained through thermal evaporation, the memory window of the order of 5.1 V was shown [20]. If one takes into account the threshold voltage (V_t_) shift, the values of the order of 1.5 V [21] or 1–3 V [22] have been reported. The simulation studies forecasted the charge carriers retention with the memory window of the order of 3 V for up to 10 years [23] with the application of high-*k* dielectric films. Thus, according to the reported results, the findings demonstrated in this work should encourage further investigations on the application of SiC-NCs in NVSM structures. It has to be underlined that a large memory window of the order of 2.7 V and 2.9 V, for the smaller and larger SiC-NCs, respectively, as well as being extrapolated to 10 years, was obtained.

**Figure 10:**
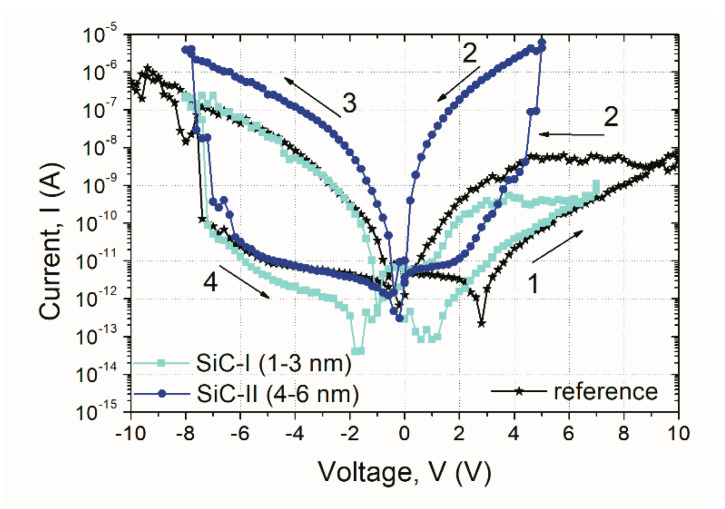
Comparison of representative resistive switching I–V curves of NC-MIM and reference MIM devices investigated in this work; the numbers indicate the sequence of measurements.

In the last part of this study, the fabricated MIM structures with embedded SiC-I and SiC-II nanoparticles were examined. The representative I–V characteristics of the sample structures are depicted in Figure 10. The results presented distinctly show that the application of SiC-NCs resulted in a higher value of the high-resistance state (HRS) to low-resistance state (LRS) ratio. It is particularly evident in the case of NC-MIM structures with SiC-II NCs (i.e., 4–6 nm). Moreover, the HRS to LRS ratio of the structure with SiC-II NCs increased compared to the device based on SiC-I NCs. It may be connected to the fact that the application of large NPs resulted in a larger local electric field, that in the case of LRS, resulted in a higher leakage current, and thus a higher HRS to LRS ratio. However, such a hypothesis needs further study as well as more statistical data, both of which go beyond the aims of this preliminary study.

In the case of NC-MIM structures with large SiC nanoparticles, the ratio between LRS to HRS was about four orders of magnitude, compared to two orders of magnitude in the case of reference structures. Furthermore, the NC-MIM structures with SiC-II NCs exhibited lower set/reset (V_set_/V_reset_) voltage values that controlled the electroforming process. As can be drawn from Figure 10, the V_set_ was ~−6.2, and ~−7.4 V, whereas the V_reset_ was ~4.8, and ~6 V for NC-MIM with SiC-II NCs and reference structures, respectively. This was due to the fact that inserted nanoparticles may enhance the local electric field [24]. In this case, SiC-NCs effectively supported the formation of current paths and the leakage filament formation under the influence of electrical polarization, thus affecting the switching properties of the memory cell. Such a finding is of a great importance for the reduction of the device’s power consumption level.

## 5. Conclusions

The technology of silicon-carbide nanoparticles has been developed and fabricated using a method is based on reactive bonding, followed by electroless wet chemical etching. The performed structural and optical characterization of fabricated nanocrystals showed that the NPs were well crystalline without the detectable presence of any amorphous or oxide phases. SiC-NCs were also incorporated into MIS and MIM memory structures. Two types of NPs with different dimensions were investigated, i.e., nanocrystals in the range of 1–3 nm (SiC-I), and 4–6 nm (SiC-II). During the fabrication of sample devices with SiC-NCs embedded in hafnium oxide thin films, SiC nanocrystals were transferred from a methanol solution. The obtained sample devices were examined throughout the stress-and-sense measurements in terms of device capacitance, flat-band voltage shift, switching properties, and retention time.

The demonstrated findings in this study have shown a significant hysteresis of the V shift induced by the program and erase voltages of the fabricated NC-MIS devices. A change of V due to the V of the order of 7.6 V and 8.6 V was demonstrated in the case of structures with SiC-I and SiC-II, respectively. The obtained charge retention for the examined structures revealed relatively high stability. Taking into account the dimensions of SiC-NCs, the memory window of the order of 2.7 V and 2.9 V for the smaller (i.e., 1–3 nm) and larger (i.e., 4–6 nm) NCs were calculated (extrapolated to 10 years) respectively. A slightly higher memory window width and higher retention loss for NC-MIS structures with SiC-II were found. This finding is related to deteriorated electrical parameters, i.e., higher Q_eff_ and D_it_ values that were proved by the analysis of high-frequency C–V characteristics. Moreover, the presented results of the analysis of electrical characteristics of the fabricated NC-MIM devices have shown the narrower set and reset operation range and a larger HRS to LRS ratio in the devices with SiC-NCs as compared to the reference devices.

The results presented in this study are promising for potential applications of SiC nanocrystals, particularly in NVSM devices, or the possible replacement of silicon–oxide–nitride–oxide–silicon (SONOS) gate-stack or RRAM structures. The presented results are original and initial, as they deal with SiC-NCs dispersed in methanol solution that were spin-coated to form an active part of the device and make the examined devices interesting for further research and technology development.
